# Monitoring Inequalities in the Health Workforce: The Case Study of Brazil 1991–2005

**DOI:** 10.1371/journal.pone.0033399

**Published:** 2012-03-27

**Authors:** Angelica Sousa, Mario R. Dal Poz, Cristiana Leite Carvalho

**Affiliations:** 1 Department for Health Systems Policies and Workforce, World Health Organization, Geneva, Switzerland; 2 Center for Population and Development Studies, Harvard School of Public Health, Boston, Massachusetts, United States of America; 3 Institute of Social Medicine, University of the State of Rio de Janeiro, Rio de Janeiro, Brazil; 4 Dental School, Pontifical Catholic University, Minas Gerais, Brazil; University of Colorado Denver, United States of America

## Abstract

**Introduction:**

Both the quantity and the distribution of health workers in a country are fundamental for assuring equitable access to health services. Using the case of Brazil, we measure changes in inequalities in the distribution of the health workforce and account for the sources of inequalities at sub-national level to identify whether policies have been effective in decreasing inequalities and increasing the density of health workers in the poorest areas between 1991 and 2005.

**Methods:**

With data from Datasus 2005 and the 1991 and 2000 Census we measure the Gini and the Theil T across the 4,267 Brazilian Minimum Comparable Areas (MCA) for 1991, 2000 and 2005 to investigate changes in inequalities in the densities of physicians; nurse professionals; nurse associates; and community health workers by states, poverty quintiles and urban-rural stratum to account for the sources of inequalities.

**Results:**

We find that inequalities have increased over time and that physicians and nurse professionals are the categories of health workers, which are more unequally distributed across MCA. The poorest states experience the highest shortage of health workers (below the national average) and have the highest inequalities in the distribution of physicians plus nurse professionals (above the national average) in the three years. Most of the staff in poor areas are unskilled health workers. Most of the overall inequalities in the distribution of health workers across MCA are due to inequalities within states, poverty quintiles and rural-urban stratum.

**Discussion:**

This study highlights some critical issues in terms of the geographical distribution of health workers, which are accessible to the poor and the new methods have given new insights to identify critical geographical areas in Brazil. Eliminating the gap in the health workforce would require policies and interventions to be conducted at the state level focused in poor and rural areas.

## Introduction

Despite the increased evidence that health workers are fundamental for improving the levels of intervention coverage and the health of the population [1]–[7] several countries still face severe shortages of health workers [3] and in many others there are large inequalities in the distribution of the health workforce within the country [8]. Both the quantity and the distribution of health workers in a country are fundamental for assuring equitable access to health services. This is particularly important as progress towards achieving MDGs targets on maternal and child mortality has slowed down by large differentials between poor and rich populations [9].

In Brazil the main problems originate from the unequal distribution of health workers within the country. The latest data from 2007 and 2009 show that most of the nurses and physicians are concentrated in the richest areas of the country [10], [11]. Despite the last two decade major health reforms implemented in Brazil seeking to enhance the local accessibility of health services and improve the health of the poor, several studies have found that poorer populations have less access to health services and worse health than richer populations [12]–[22].

While there have been great improvements in decreasing inequalities in several health indicators and improving the socioeconomic conditions of the population [10], [20], [23], social deprivation and lack of access to health services impede 16 million people to come out of extreme poverty. A large majority of the extreme poor lives in the Northeast Region and in rural areas [24]. It is therefore crucial to measure inequalities in the distribution of the health workforce and account for the sources of the inequalities to identify critical geographical areas. Although differences in the densities of health workers between urban-rural stratum or regions have been documented few studies have been undertaken to measure and monitor inequalities in the distribution of health workers across lower geographical levels of desegregation throughout the country [11] e.g. minimum comparable areas.

In addition, new evidence has highlighted that the methods to properly measure inequalities in the distribution of the health workforce in countries have not been fully used in this domain of research. An innovative study, Anand S. 2010, shed light on this issue and found that from all the inequality indices proposed in the economic literature, three indices have been shown to be more appropriate to measure inequalities in the distribution of the health workforce and account for the sources of the inequalities in a country: the Gini, the Theil L and T indices [8].

The aim of this paper is to apply these new methods to measure the changes in the inequalities in the distribution of the health workforce at sub-national level in Brazil and account for the sources of the inequalities in a period where major health reforms have been implemented to identify whether policies have been effective in decreasing the inequalities in the density of health workers among the poorest and richest areas between 1991 and 2005. This study is particularly important as one of the main priorities of the Brazilian government is to eradicate extreme poverty by 2014, a recent programme was launched to provide opportunities in terms of education, health and employment to help lift people out of extreme poverty [24].

## Materials and Methods

We used data from the two population Census, 1991 and 2000, [25], [26] for the 4,267 Minimum Comparable Areas (MCA) and from Datasus 2005 [27], [28] for the 27 states to investigate inequality trends in the distribution of the health workforce at sub-national level in Brazil. Minimum Comparable Areas (MCA) are the smallest geographical unit comparable across time.

The number of health workers per capita, were extracted from the 1991 and 2000 population Censuses microdata using sample weights to obtain population estimates for the 4,267 MCA. Four categories of health workers; in the form of densities per 1000 population; are used in this analysis to investigate inequalities in the distribution of the health workforce and account for the sources of these inequalities in Brazil: physicians; nurse professionals; nurse associates (defined as the number of people who reported been a technicians, auxiliaries of nursing, assistants nursing, practical midwives and similar by occupation); and community health workers.

We additionally extracted the densities of the four categories of health workers at state level for 2005 from Datasus 2005 -Conselhos profissionais (Ministério da Saúde/SGTES/DEGERTS/CONPROF) and Sistema de Informação da Atenção Básica (SIAB/Ministério da Saúde)- to calculate the growth rates between 2000 and 2005 for each category of health workers. We then extrapolated the growth rates at state level to the 2000 Census data to calculate the densities of physicians; nurse professionals, nurse associates and community health workers for the 4,267 MCA in 2005.

We also used data on the proportion of population below the poverty line to characterize the MCA by socioeconomic status. These data were obtained from the Institute of Applied Economic Research and were estimated from the 2000 population Census of Brazil [29].

### Methods

Anand S. 2010 identified three inequality indices which are more appropriate to measure inequalities in the distribution of health workers and account for the sources of the inequalities in a country: 1) the Gini coefficient, as it is a well known measure to account for inequalities in the distribution of other indicators such as household income, however it has the disadvantage that it can not be decomposed to account for the sources of the inequalities; 2) the Theil L, as it can be decomposed in inequalities within and across sub-national areas in a country; and 3) the Theil T index, as it can also be decomposed as the Theil L index; but it has the advantage to measure inequalities when a sub-national unit does not have health workers, e.g. when there are no doctors in a MCA -further details on the methods are reported elsewhere [8]-.

We therefore applied the Gini and the Theil T to investigate inequality trends in the densities of physicians; nurse professionals; nurse associates; and community health workers across MCA in Brazil for 1991, 2000 and 2005. We measured the Theil T rather than the Theil L because in Brazil numerous MCA do not have health workers. We additionally measured the bias that could be introduced when MCA with no health workers are not accounted in the Theil T estimation. The Theil T is also measured with MCA clustered by states, poverty quintiles and urban-rural stratum to account for the sources of inequalities and identify critical geographical areas. The analysis was performed using STATA 11 [30], [31]

## Results

In general we found that between 1991 and 2005 there has been an increase on the density of health workers per 1000 population in Brazil (see Table 1). The national density of physicians plus nurse professionals per 1000 population increased from 1.38 in 1991 to 1.49 in 2000 to 1.80 in 2005. A sharper growth is found between 1991 and 2005 in the density of nurse associates plus community health workers which increased from 3.42 in 1991 to 4.53 in 2000 to 6.96 in 2005. However it is interesting to point out that in 2005 the total density of nurse professionals is the lowest with 0.39 nurses per 1000 population and that the density of nurse associates is the highest with 5.5 nurses per 1000 population.

**Table 1:** Descriptive statistics.

	1991					2000					2005				
Densities	mean	p50	sd	min	max	mean	p50	sd	min	max	mean	p50	sd	min	max
Physicians	1.17	0.62	1.35	0.00	7.80	1.17	0.66	1.31	0.00	9.24	1.42	0.83	1.54	0.00	10.35
Nurse professionals	0.21	0.08	0.28	0.00	7.15	0.32	0.20	0.34	0.00	6.65	0.39	0.24	0.43	0.00	7.78
Nurse associates	3.06	3.07	1.80	0.00	23.89	3.56	3.58	1.75	0.00	24.95	5.50	5.39	3.12	0.00	32.69
Community health workers	0.35	0.21	0.51	0.00	12.51	0.96	0.80	0.78	0.00	16.83	1.45	1.23	1.10	0.00	23.06
Skilled HW	1.38	0.79	1.57	0.00	8.92	1.49	0.88	1.58	0.00	10.77	1.80	1.07	1.87	0.00	12.01
Not skilled HW	3.42	3.50	1.81	0.00	23.89	4.53	4.56	1.72	0.00	24.95	6.96	6.75	3.22	0.00	34.25

Sources: Data were extracted from the population Census 1991 & 2000. We also used data at state level from Datasus 2005 -Conselhos profissionais (Ministério da Saúde/SGTES/DEGERTS/CONPROF) and Sistema de Informação da Atenção Básica (SIAB)-,

Note: Skilled (HW) Health Workers is defined as physicians and nurse professionals and not skilled Health Workers as community health workers and nurse associates. Densities are calculated per 1000 pop.

A number of policies have been implemented to increase the number of health workers between 1991 and 2005. For example, PROFAE - Projeto de Profissionalização dos Trabalhadores da Área de Enfermages was created in 2000 with the aim to increase the supply of nurse associates in the country. The main target was to train 225 000 mid-level nurses and 12,000 teaching nurses. Since the project was implemented even more providers have been trained, the latest data from 2008 shows that 235,172 nurses have graduated as nurse associates [32], [33].

For this analysis we grouped physicians together with nurse professionals as in Brazil these categories of health workers are considered as skilled health workers as they have university education, while nurse associates and community health workers have high school and vocational courses and therefore are considered as non skilled health workers.

**Figure 1:**
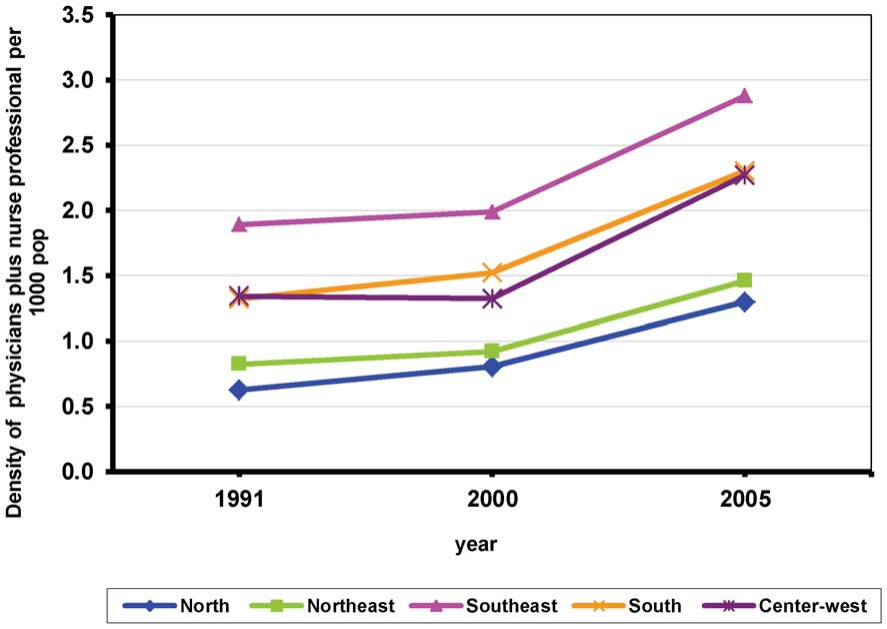
Trends of the density of physicians plus nurse professional per 1000 pop by region in Brazil 1991–2005. Author's calculation using data of the population Census 1991 & 2000 and Datasus 2005 -Conselhos profissionais (Ministério da Saúde/SGTES/DEGERTS/CONPROF). Note: X axis = year. Y axis = density of physicians plus nurse professionals per 1000 pop. Blue diamond = North Region. Green square = Northeast Region. Purple cross = Centre West Region. Yellow cross = South Region. Pink triangle = Southeast Region.

Although there has been a very important increase in the number of health workers the absolute differences show that there is great variability in the densities of health workers across regions, states and MCA in the period analysed. Figure 1 shows the differences in the trends of the densities of physicians plus nurse professionals by region between 1991 and 2005. We found that the richest Regions (South and Southeast) have more skilled health workers and at the same time have experienced a sharper growth in the densities of these categories of health workers between1991 and 2005 than the poorest Regions (North and Northeast). We also found great differences across MCA, 61% of the MCA did not have physicians plus nurse professionals in 1991 and 53% in 2005, compare to some areas that had densities above 9 per 1000 pop.

We applied the Gini, and the Theil T indices to investigate inequality trends in the distribution of health workers across MCA, for 1991, 2000 and 2005 (see Table 2). In general, we found that the overall inequalities in the distribution of physicians, nurse professionals, nurse associates and community health workers have decreased between 1991 and 2000. However, between 2000 and 2005, we found that inequalities have increased in the distribution of nurse professionals and nurse associates, have remained the same for the density of physicians and have steadily decreased over time for community health workers. We nevertheless found that physicians and nurse professionals are the categories of health workers which are the most unequally distributed across MCA. The Gini for nurse professionals in 1991 is .66, in 2000 is .57 and in 2005 is .59 while the Gini for physicians in 1991 is .60, and is .58 in 2000 and 2005.

**Table 2:** Inequalities in the distribution of the health workforce and the sources of the inequalities by category of health workers in Brazil 1991–2005.

	1991					2000					2005				
Densities	Gini	Theil T	Theil within (%)	Theil between (%)	bias (%)	Gini	Theil T	Theil within (%)	Theil between (%)	bias (%)	Gini	Theil T	Theil within (%)	Theil between (%)	bias (%)
Physicians	0.60	0.64	0.38 (60)	0.26 (40)	0.28 (44)	0.58	0.60	0.36 (60)	0.24 (40)	0.25 (41)	0.58	0.59	0.50 (84)	0.10 (16)	0.25 (42)
Nurse professionals	0.66	0.85	0.65 (76)	0.20 (24)	0.61 (71)	0.57	0.61	0.47 (77)	0.14 (23)	0.39 (64)	0.59	0.65	0.58 (89)	0.07 (11)	0.39 (60)
Nurse associates	0.33	0.19	0.12 (64)	0.07 (36)	0.03 (16)	0.28	0.13	0.08 (63)	0.05 (37)	0.01 (8)	0.30	0.16	0.11 (73)	0.04 (27)	0.01 (7)
Community health workers	0.63	0.75	0.68 (90)	0.07 (10)	0.32 (42)	0.42	0.30	0.23 (75)	0.08 (25)	0.05 (17)	0.38	0.26	0.21 (83)	0.04 (17)	0.05 (20)
**Poverty quintiles**															
Skilled HW	0.59	0.62	0.38 (60)	0.25 (40)	0.26 (42)	0.55	0.54	0.33 (61)	0.21 (39)	0.19 (35)	0.55	0.54	0.34 (63)	0.20 (37)	0.19 (35)
Not skilled HW	0.29	0.15	0.11 (73)	0.04 (27)	0.02 (12)	0.21	0.08	0.06 (82)	0.01 (18)	0.00 (4)	0.24	0.10	0.09 (85)	0.02 (15)	0.00 (3)
**Stratum (Uban-rural)**															
Skilled HW	0.59	0.62	0.48 (77)	0.15 (23)	0.26 (42)	0.55	0.54	0.46 (85)	0.08 (15)	0.19 (35)	0.55	0.54	0.46 (85)	0.08 (15)	0.19 (35)
Not skilled HW	0.29	0.15	0.12 (79)	0.03 (21)	0.02 (12)	0.21	0.08	0.07 (88)	0.01 (12)	0.00 (4)	0.24	0.10	0.09 (91)	0.01 (9)	0.00 (3)
**States**															
Skilled HW	0.59	0.62	0.51 (81)	0.12 (19)	0.26 (42)	0.55	0.54	0.45 (83)	0.09 (17)	0.19 (35)	0.55	0.54	0.47 (83)	0.07 (17)	0.19 (35)
Not skilled HW	0.29	0.15	0.12 (80)	0.03 (20)	0.02 (12)	0.21	0.08	0.07 (92)	0.01 (8)	0.00 (4)	0.24	0.10	0.08 (74)	0.03 (26)	0.00 (3)

Sources: Author's calculation using data of the population Census 1991 & 2000, Datasus 2005 -Conselhos profissionais (Ministério da Saúde/SGTES/DEGERTS/CONPROF) and Sistema de Informação da Atenção Básica (SIAB/Ministério da Saúde)-, and the Institute of Applied Economic Research (IPEA).

Note: Skilled (HW) Health Workers is defined as physicians and nurse professionals and not skilled Health Workers as community health workers and nurse associates. Densities are calculated per 1000 pop.

The main advantages of the Theil T are that it can be decomposed in inequalities within and across sub-national areas to identify the sources of the inequalities in a country and it can also be measured when MCA do not have health workers. We then measured the percentage of underestimation if MCA with no health workers were not included in the calculations and found that 42% of overall inequality in the distribution of physicians and 60% in the distribution of nurse professionals in 2005 is due to MCA with no health workers.

We clustered MCA by different population subgroups to identify the sources of the inequalities. We first started with the partition of MCA by state and found that most of the overall inequalities in the distribution of health workers across MCA are due to inequalities within states which account for around 60% to overall inequality and this has not changed over time. Moreover we found that in general within state inequalities have increased in time. For both physicians plus nurse professionals inequalities within states accounted for 81% of the overall inequality in 1991 and it increased to 83% in 2005. The opposite tendency is found for nurse associates plus community health workers where the percentage of within states inequality to overall inequality was 80% in 1991, 92% in 2000 and it decreased to 74% in 2000.

We also measured inequalities by poverty quintiles and found contrary to what was expected that inequalities between the poor and rich are not that high; most of the total inequality is explained by inequalities within poverty quintiles and this is encountered for the three years analysed. For physicians plus nurse professionals the between poverty quintile inequalities accounts for only 37% of overall inequality and most of the overall inequality (63%) is due to inequalities within poverty quintiles. In fact, the highest inequalities are found within the poorest quintile. For nurse associates plus community health workers the percentage contribution of between poverty quintiles to overall inequality is even smaller and has decreased in the period analysed, from 27% in 1991 to 15% in 2005. Similarly, Anand S. 2010, found that inequalities between income deciles explain only 20% of the total inequality in rural counties in China.

In the partition of MCA by urban-rural stratum we found that most of the overall inequality in the distribution of physicians plus nurse professionals across MCA is due to inequalities within urban-rural stratum, which account for around 77% to overall inequality in 1991 and this percentage has increased to 85% in 2005. The highest inequalities are found within the rural stratum. For nurse associates plus community health workers we found the same trend, the within urban-rural stratum accounts for 79% of the overall inequality in 1991 and this percentage increased to 91% in 2005.

**Figure 2:**
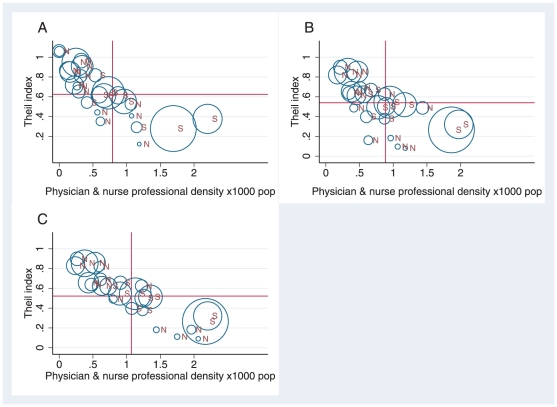
Relationship between the level of inequality and the density of physicians plus nurse professionals per 1000 pop by state and region in Brazil 1991–2005. Author's calculation using data of the population Census 1991 & 2000 and Datasus 2005 -Conselhos profissionais (Ministério da Saúde/SGTES/DEGERTS/CONPROF). Note: X axis = density of physicians plus nurse professionals per 1000 pop. Y axis = Theil T index. Panel A = relationship for the year 1991. Panel B = relationship for the year 2000. Panel C = relationship for the year 2005. Each dot represents a state. The horizontal and vertical line represents the national averages of both variables. S refers to states in the Southern Regions and N refers to states in the Northern Regions. Area of the symbol proportional to state's population.

Figure 2 shows the relationship between the level of inequality and the density of both physicians plus nurse professionals by state and region for 1991 (A), 2000 (B) and 2005 (C). The horizontal and vertical lines in A, B and C represent the national averages of both variables. We found that the majority of the states which belong to the poorest regions (North and Northeast Regions) experienced the highest shortage of health workers; with densities below the national average; and at the same time have the highest inequalities in the distribution of physicians plus nurse professionals; with inequalities above the national average in the three years analysed. These states are also among the poorest states with the highest population density. In contrast we found that states in richer regions (South, Southeast and Central West Regions) have the lowest inequalities (below the national average) and the highest numbers of physicians plus nurse professionals per 1000 pop (above the national average).

Although most of the poorest states have high inequality in the distribution of physicians and nurse professionals it is important to highlight that some states have attained very important progresses in decreasing the level of inequality and decreasing the shortage of physicians and nurse professionals per 1000 pop. For example, the states of Amapá which has decreased the level of inequality from .41 in 1991 to .11 in 2005 and Alagoas which has decrease the level of inequality from 1.06 to .69 and has also increased the density of physicians plus nurse professionals. Progress in these states are mainly due to a higher increase in their financial resources to human resources for health in the period analysed. The state of Amapa experienced an increase in all health resources as between 1991 and 2005 it benefited from the highest increase (more than three times) of federal funds allocated to health. It is important to highlight that in 1991, it was also the sate which received the least resources from the federal governmentt. In the case of Alagoas progress towards decreasing inequalities are mainly due to the fact that it is among the states with the highest increased in the allocation of health expenditure to human resources for health an increase of more than 1.3 times between 1991 and 2005 [34].

## Discussion

We found that between 1991 and 2005 there have been great improvements in increasing the availability of health workers in Brazil. However, despite these improvements, we found that the overall inequalities in the distribution of health workers have increased between 2000 and 2005, except for community health workers which have experienced a decrease over time. Physicians and nurse professionals are the categories of health workers which are more unequally distributed across MCA.

Despite the efforts made by the Brazilian government to enhance the local accessibility of health services particularly among the poor, we found that the poorest states -from the North and Northeast Regions- experienced the highest shortage of health workers and at the same time have the highest inequalities in the distribution of skilled health workers such as physicians and nurse professionals and this problem has not changed over time. The majority of the staff in these geographical areas are nurse associates and community health workers which have less skills and education.

We also found that most of the overall inequalities in the distribution of health workers across MCA are due to inequalities within states, within poverty quintiles and within rural-urban stratum and this has not changed over time. Thus policies and interventions should be directed to increase the availability of health workers in poor and rural areas at state level.

This study has highlighted some critical issues in terms of the geographical distribution of skilled health workers and the new methods have given new insights to identify critical geographical areas in Brazil. Although geographical areas with more health workers are more likely to have better population health further analysis should be undertaken to compare health outcomes between areas with varying levels of skilled health workers to determine if there are direct measurable effects of health workers supply and skilled mix composition in the health of the population. This type of analysis is essential to advance the knowledge on the type of health system policies that could have a direct effect in improving the health of the poorest population. In this critical point in time when we are approaching the 2015 deadline to attain the Millennium Development Goals (MDGs) targets, it is crucial that countries share experiences in policies that have been effective in improving the health of the poorest populations.

The conclusions drawn from this study should take into consideration the limitations of the data. Although municipality is the geographical unit where decisions are made after the decentralization reform, we did not used municipalities in this analysis as it is a geographical unit that is not comparable over time. This is because a number of municipalities have been created between 1991 and 2000, from 4,491 to 5,507. To overcome this problem we used data for MCA (4,267) as it is the smallest geographical unit that is comparable over time. We produced estimates for the MCA for 2005 from the 2000 population census as the censuses are the sources of information with more relive data on the variables used in this study for the minimum comparable areas. We used the information from Datasus to produce the 2005 data as we compared the densities of physicians and nurse professionals from other sources at state level and found that Datasus is the only source that produces similar densities of health workers than the population Census. Since we used data at state level to extrapolate the densities of the different type of health workers at MCA in this study it is probable that the densities at the MCA level may differ from the current numbers.

The results found in this study are in line with recent studies, which showed great inequalities in the distribution of health workers across region using more recent data from 2007 [10]. Given the last years expansion of investment in the poorest regions -North and Northeast-, with strategies such as the Health family Programme to increase the availability of family health teams and increase coverage of health services, [35], further analyses using the methods presented in this paper will be key to monitor the impact of these policies to decrease inequalities in the distribution of health workers at sub-national level. This will be possible when the more recent Census data will be available in 2012.
